# Personality, density and habitat drive the dispersal of invasive crayfish

**DOI:** 10.1038/s41598-021-04228-1

**Published:** 2022-01-21

**Authors:** Shams M. Galib, Jingrui Sun, Sean D. Twiss, Martyn C. Lucas

**Affiliations:** 1 Department of Biosciences, University of Durham, Stockton Road, Durham, DH1 3LE UK; 2 Department of Fisheries, University of Rajshahi, Rajshahi, 6205 Bangladesh; 3 Yunnan Key Laboratory of International Rivers and Transboundary Eco-Security, Yunnan University, Kunming, 650091 China; 4 Institute of International Rivers and Eco-Security, Yunnan University, Kunming, 650091 China

**Keywords:** Ecology, Invasive species, Behavioural ecology

## Abstract

There is increasing evidence that personality traits may drive dispersal patterns of animals, including invasive species. We investigated, using the widespread signal crayfish *Pacifastacus leniusculus* as a model invasive species, whether effects of personality traits on dispersal were independent of, or affected by, other factors including population density, habitat, crayfish size, sex and limb loss, along an invasion gradient. Behavioural traits (boldness, activity, exploration, willingness to climb) of 310 individually marked signal crayfish were measured at fully-established, newly-established and invasion front sites of two upland streams. After a period at liberty, recaptured crayfish were reassessed for behavioural traits (newly-established, invasion front). Dispersal distance and direction of crayfish movement, local population density, fine-scale habitat characteristics and crayfish size, sex and limb loss were also measured. Individual crayfish exhibited consistency in behavioural traits over time which formed a behavioural syndrome. Dispersal was both positively and negatively affected by personality traits, positively by local population density and negatively by refuge availability. No effect of size, sex and limb loss was recorded. Personality played a role in promoting dispersal but population density and local habitat complexity were also important determinants. Predicting biological invasion in animals is likely to require better integration of these processes.

## Introduction

Dispersal is a fundamental ecological process, necessary for the persistence of almost all organisms in patchy environments^1^. Determining the mechanisms that underpin dispersal is key to themes such as invasion ecology, metapopulation ecology and range responses to climate change^2,3^. Dispersal can be influenced by a variety of factors, including morphological^3,4^, physiological^3,4^ and behavioural^4–6^ phenotypes. Within populations, individuals often differ consistently in their behaviours across time and contexts^7–10^. Those consistent inter-individual differences, often regarded as individual behavioural types or personalities^11^, can play a key role in determining how individuals interact with their environment^12^ and consequently, can be significant drivers of population dynamics, with impacts on a range of life history stages^13,14^.

The effect of personality on invasion dynamics, particularly dispersal, has been identified as a potentially important driver of invasion success in animals^13,15,16^. Although invasive species may generate some perceived benefits (e.g. non-native European catfish providing recreational angling opportunities for a top predator^17^), they are mostly recognised for large-scale biological and ecological consequences^1,18,19^, including severe impacts on native species and ecosystems, and are important agents of global change^20,21^, causing severe economic damage^22^. Therefore, understanding mechanisms underpinning dispersal in non-native species is important.

Personality traits are often correlated with each other, comprising ‘behavioural syndromes’^11^ and therefore multiple facets of an individual’s interaction with its environments may be dictated, in part, by these behavioural correlations. Personality does not preclude behavioural plasticity, but evidence of consistent individual differences in behaviour does imply that individuals can only modify behaviour within certain bounds, such that rank order differences in behaviour are maintained across contexts or situations^23^. Whether personality is heritable^24^, or a product of early developmental environment^25,26^, the existence of consistent individual differences in behavioural traits can have fundamental impacts on population ecological processes^8,10,27,28^, as this difference between individuals can be important for successful resource acquisition and mating.

Dispersal and range expansion of a population is influenced by several personality traits. Enhanced exploration and activity is often linked with increased fitness and thus, more exploratory/active individuals are expected to play a key role in range expansion by dispersing further^12^ but often expose themselves to greater predation risks for resource acquisition through dispersal^29^. Boldness can also positively affect the spread of a population^30^. Recent literature has suggested that the presence of individuals that are bold, more asocial and active helps invasive populations to spread further^30^ and personality-biased dispersal could be expected on the invasion front^13,15^. Thus, personality-dependent dispersal might be an important factor in determining success of biological invasion, but few studies have focused on this issue so far^13,15,31^. However, the role of personality in determining invasiveness can be unclear^32^. Some studies suggest that individuals on the invasion front, those leading the range expansion, are more aggressive or active than their counterparts inhabiting established areas^13,33^. Conversely, other studies show less aggressive or less bold individuals lead the invasion^15,34^, perhaps because risk averse individuals could invest more energy in reproduction under relaxed intraspecific competition at the invasion front^34^. These seemingly contradictory results suggest that invasion dynamics may be influenced by other factors in complex interactions with personality traits^35–37^. Most studies on the role of behavioural types in invasion by non-native species ^15,34^ were carried out in laboratory environments and their results may not be representative of processes in the natural environment, where context may vary between species, populations, personality types, and over time^38^. Therefore, a better understanding is needed of the complexity of range dynamics with respect to personality in the wild^9,39^ within and between populations^5^. In animal populations, evidence of temporally consistent, cross-contextual patterns of personality-dependent movement remains rare^40,41^.

Using invasive crayfish in streams as a study model, we hypothesized that dispersal tendency is affected by personality traits which remain consistent over time and form a behavioural syndrome. We predicted that bold crayfish, with a high exploratory drive, disperse further in nature. We also hypothesized that dispersal tendency is affected by habitat quality and therefore predicted that local population density and refuge availability alter dispersal rate positively and negatively respectively, mediated by competitive behaviour^42^. Therefore, we measured five behavioural indicators of boldness and exploration in signal crayfish (*Pacifastacus leniusculus*), a well-known freshwater invasive species^43^. We investigated whether behavioural traits indicative of boldness and exploration in signal crayfish were consistent over time, and whether these traits constituted a behavioural syndrome. We investigated the pattern of dispersal in relation to these behavioural traits along an invasion gradient. We also determined the influences of crayfish sex, size, limb loss, population density and habitat characteristics on dispersal, to better understand underlying invasion dynamics in relation to behavioural traits.

## Methods

### Study sites

**Figure 1:**
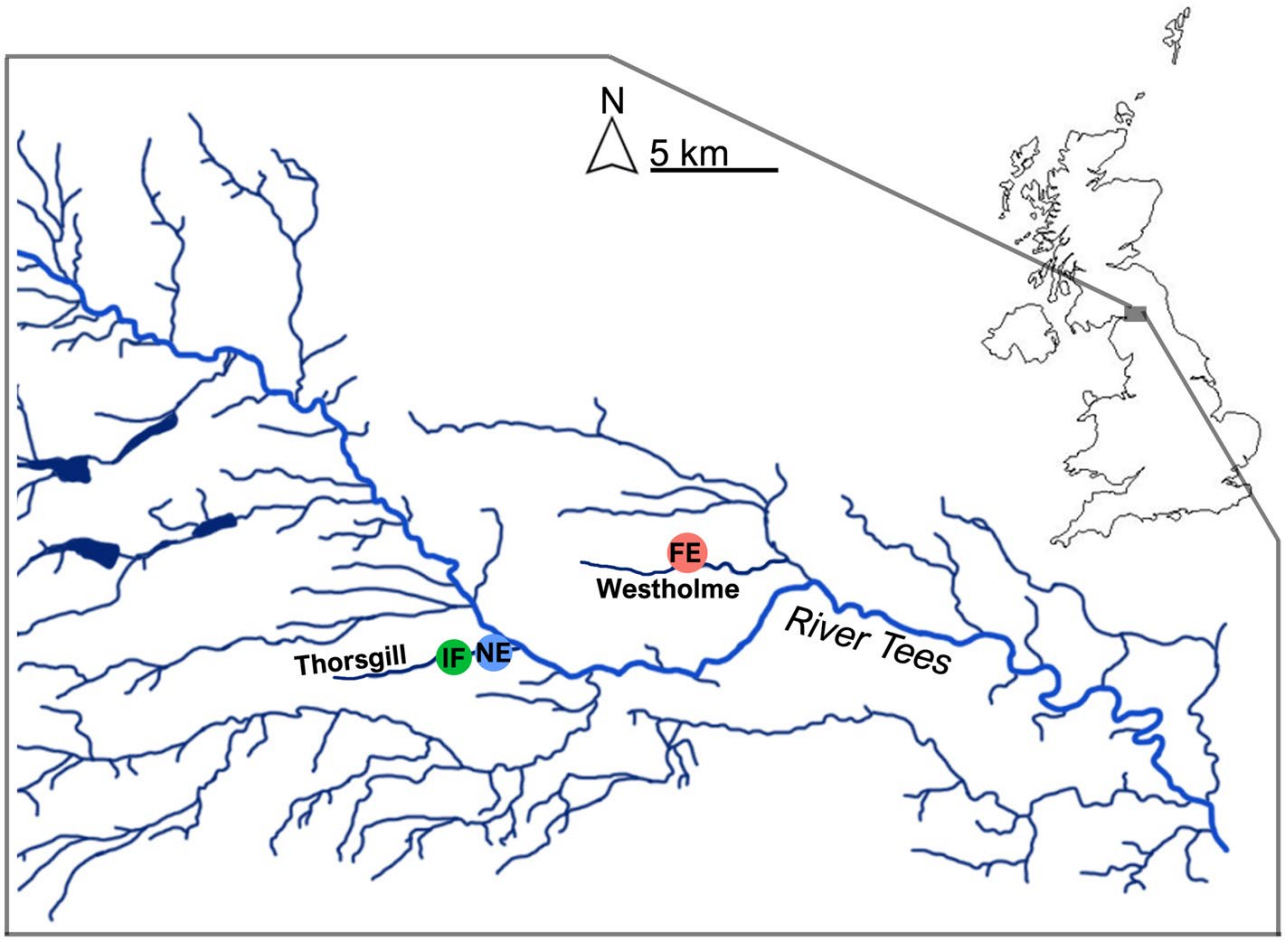
Map of the study locations of invasive crayfish dispersal in the River Tees catchment, northeast England. *FE* fully-established, *NE* newly-established, *IF* invasion front.

This study was conducted in two tributaries of the River Tees, northeast England. Signal crayfish stocked at a single site in the 1980s spread along the Tees and have invaded multiple tributaries in an upstream direction^44^. Experiments were carried out from 7 August to 28 September 2017 in Westholme Beck (54° 33′ 26.3″ N 1° 47′ 53.0″ W, Fig. 1) at which signal crayfish are fully-established and at high density (mean minimum crayfish density ± SD, 2.2 ± 1.9 m^−2^ based on area sampling, see Supplementary Methods for further detail). Signal crayfish invaded Westholme Beck from the Tees between 1995 and 2000 (M.C. Lucas, pers. obs.). Fieldwork was also carried out in Thorsgill Beck (54° 31′ 53.5″ N 1° 54′ 46.3″ W, Fig. 1) between 6 August and 20 September 2018, where signal crayfish are newly-established (invaded from Tees 2011–2012^44^) and at lower density (1.1 ± 0.7 m^−2^). We also sampled the invasion front of Thorsgill (density 0.25 ± 0.3 m^−2^) as dispersal biology at the invasion front can differ from that in the core^45^. Experiments were conducted at the same time of year and sites had very similar physico-chemical characteristics, including photoperiod, temperature, flow conditions, pH and oxygen (Table 1). Terrestrial predators of crayfish at both sites, grey heron (*Ardea cinerea*) and Eurasian otter (*Lutra lutra*), were rarely spotted during the study; otter spraint and regurgitated heron pellets were present but infrequent at both sites. Small (< 20 cm) brown trout (*Salmo trutta*) and bullhead (*Cottus perifretum*) were the main stream fishes, but crayfish size used in this study (reflecting the dominant size class involved in upstream invasion^46^) were larger than the gape size of these fishes.

**Table 1:** Measured habitat/environmental characteristics of the study sites (as Mean ± SD or range).

Characteristics	Westholme Beck (2017)	Thorsgill Beck (2018)
Wetted width (m)	2.5 ± 0.75	3.7 ± 1.1
Water depth (cm)	13.9 ± 9.4	12.8 ± 5.0
pH	8.1–8.5	8–8.2
Dissolved oxygen (mg L^−1^)	9.4–11.5	9.9–11.4
Water flow velocity (m s^−1^)	0.05–0.8	0.05–0.6
Water temperature (°C)	15.1 ± 0.8 (13.6–16.1)	15.5 ± 0.8 (14.2–16.9)
Water level (cm, during behaviour measurements)	10.76 ± 4.2	10.37 ± 6.00
High flow event during study	No	No

Measurements for width, depth and flow velocity were made during crayfish recapture surveys in September of each year, while measures of pH, water temperature and dissolved oxygen were made from early August to late September. Measurement method details are available in the supplementary methods.

### Collection of signal crayfish

Signal crayfish for behavioural typing and marking were collected randomly by hand-net searching within stream segments during daytime. It is rare to find wild signal crayfish in shallow upland streams outside refuges by day (S. Galib, pers. obs.) and in this study all were collected from instream refuges, almost entirely under non-embedded cobbles and boulders. In Westholme 130 signal crayfish (> 20 mm carapace length [CL]) were collected from the central 60 m of the 1-km study site (Table 2). In Thorsgill 180 crayfish (Table 2) were collected from similar-length reaches of the invasion front and newly-established locations (90 from each site). Capture location was recorded relative to fixed 5 m markers along stream banks and by GPS (accuracy ± 3 m), so that crayfish could be released at their capture locations. After collection, crayfish were held individually in 15 L aerated river water tanks, periodically refreshed, and placed in the stream for 3 h acclimatisation prior to behavioural testing. Three cobbles from the stream were provided in each tank for shelter. Further detail is given in the Supplementary Information.

**Table 2:** Numbers of marked, and recaptured, signal crayfish, as well as sex ratios and summary statistics for carapace length.

Stream and site	Total crayfish marked			Recaptured crayfish		
	N	Sex ratio (M:F)	Carapace length (mm; Mean ± SD and range)	N	Sex ratio (M:F)	Carapace length (mm; Mean ± SD and range)
Westholme, Fully-established	130	1:1.20	33.1 ± 5.6 (23.0–55.6)	41	1:0.58	31.8 ± 4.6 (23.4–48.2)
Thorsgill, Newly-established	90	1:0.80	35.7 ± 6.4 (24.5–59.1)	32	1:1.13	35.2 ± 5.7 (24.5–47.5)
Thorsgill, Invasion front	90	1:0.58	38.6 ± 7.9 (25.9–59.8)	25	1:0.67	39.9 ± 7.8 (31.2–59.8)
Overall	310	1:0.87	35.44 ± 6.9 (23.0–59.8)	98	1:0.78	35.24 ± 6.9 23.4–59.8

### Assessment of crayfish activity, exploration and climbing tendencies

Behavioural tests were undertaken on the bankside, in deep shade during daytime^47^ on the day of capture. The experimental arena, half-filled with stream water, was surrounded by black curtains to minimise visual disturbance. Crayfish were tested at different times but there was no effect of time of day on behavioural traits measured (LMMs: *F* = 0.48–1.11, *p* = 0.366–0.866). Therefore, although crayfish are largely nocturnal^48^, these tests provided a standardised assay of individual differences in behaviour. The first tests measured crayfish activity, distance moved, exploration and climbing in a rectangular white plastic tub (52 cm long, 34 cm wide, 25 cm high) with a 2 × 2 cm grid on the bottom, but otherwise devoid of physical features. This test is essentially a standard open-field test, conducted in a novel environment^15^. A crayfish was transferred to a shelter (PVC half-pipe, closed at ends), always located in the same corner of the arena. After 10 min for crayfish acclimation, the shelter was removed and the crayfish’s behaviour recorded for 20 min by a camera (GoPro Hero-4) located above the tank. The arena was rinsed and refilled between tests to avoid potential effects of odours released by previously tested crayfish.

Videos were exported as image stacks (one frame per second) using the ‘ffmpeg’ application (https://ffmpeg.org) and imported into ImageJ (version 1.52a) where the crayfish’s position (position of tip of rostrum; x–y coordinates) was tracked over the 20 min assay. These data were imported into R^49^ and total distance moved during arena exploration was calculated for each crayfish as cumulative distance moved between each image. Activity was measured as the total number of seconds the crayfish was in motion, by deducting the total duration of time crayfish remained sedentary from total duration, i.e. 1200 s. Exploration was quantified as the percentage of unique grid squares touched by tip of the rostrum during the test. Videos were decoded to provide measures of total distance moved, time in motion (activity) and area explored. Climbing, a trait that could be important for walking arthropods, was defined as when the crayfish was active, with its body against the tank wall at an angle of 45–90° from horizontal. The total time spent trying to climb the sides of the tank was recorded.

### Assessment of boldness through threat response

After the exploration/activity/climbing test was completed, the tail of each crayfish was touched gently using a thin rod, from behind. This test was designed to mimic the threat of a predator in natural environments and crayfish responded in two ways; either tail-flipping (rapid contraction of the abdomen propelling the crayfish backwards) or raising their claws^50^. Crayfish were categorised into two groups representing ‘boldness types’, depending on response^51^; (1) shy (tail-flipping, retreating individuals), and (2) bold (individuals who raised their claws). We also recorded a ‘boldness score’. For shy individuals, this score was the combined duration of tail-flipping and subsequent stationary position before they started to move again. For bold individuals, this score was the total duration from initiation of claw raising to when the claws were lowered. We denoted bold individuals’ scores (i.e. duration recorded) as ‘positive’ and shy individuals’ scores as ‘negative’^52^ to generate a spectrum of bold-shyness. Although we measured boldness immediately after the open-field test, prior walking/climbing would not have caused bias in the subsequent tendency or otherwise for tail flipping, as the two locomotor behaviours use different muscle sets, and walking is primarily aerobic whereas a tail flip escape response is anaerobically-fuelled; i.e. they are largely physiologically independent^53,54^. Also, before conducting this experiment in the field we carried out a pilot experiment in the laboratory (in 2018) where we tested boldness both as a part of a continuous test (i.e. 15-min walking followed by a boldness test) and as a separate test of the same individuals after 24 h (*n* = 26). We did not find any significant difference in boldness score between tests (Mann–Whitney *U* test: *W* = 326, *p* = 0.833).

### Measurement of crayfish condition and dispersal in natural environment

We employed capture-mark-recapture in a standardized manner to estimate dispersal tendency as commonly employed for macroinvertebrates^55^. After behavioural tests were completed, carapace length (Vernier scale), body mass (+/− 0.001 g), sex and appendage condition (loss or damage to claws, legs; yes/no) of each individual were recorded. Crayfish were marked individually using Visible Implant Elastomer (VIE; Northwest Marine Technology, Inc., Shaw Island, WA^56^). Crayfish were released at their capture locations and left at liberty, without further disturbance until the recapture survey.

At Westholme (fully-established) and Thorsgill (newly-established/invasion front) recapture surveys commenced one month after release of the last crayfish (mean and SD of duration at liberty per crayfish recaptured was 34.1 ± 4.4 days). One km of stream (500 m upstream, 500 m downstream from midpoint of crayfish releases) was surveyed (hand-net searching by day; 2–3 experienced persons) by dividing the whole study length into 200 5-m long sections. Resurveying took two weeks in each stream and progressed outwards from the midpoint in each study site. Further detail is given in Supplementary Methods. All crayfish recorded originated from daytime refuges (primarily cobbles/boulders). Crayfish captured in each 5-m section were counted, measured, sexed and inspected for VIE marks. Each recaptured crayfish was identified, reweighed, measured and any appendage damage recorded. Dispersal direction (upstream or downstream) and distance from the release point was recorded. For each recaptured crayfish we computed the daily dispersal distance by dividing total distance moved by the number of days between release and recapture dates.

### Consistency in behavioural traits

At Thorsgill (newly-established/invasion front sites), the behavioural tests described above were repeated on recaptured crayfish. This allowed us to test if individuals exhibit repeatable measures for activity, distance moved, exploration, climbing and boldness, and whether any relationship between these behavioural measures also persists over time, indicative of a behavioural syndrome^15^.

### Measuring habitat and population density characteristics

During recapture surveys habitat characteristics (water depth, velocity, wetted width, refuge availability) were recorded within the 5-m sections of each study site. Water depth and velocity were recorded at 25%, 50% and 75% width positions of the streams across transects at the downstream end, middle and upstream end of each 5-m section. Refuge availability is a crucial habitat factor for crayfish^42^ and in upland streams like ours it is mostly provided by unembedded cobbles and boulders^42^ without instream macrophytes, and almost no crayfish holes in the bank or exposed silt beds. In each 5-m section an index of refuge availability was determined by measuring the area of unembedded streambed cobbles/boulders of ≥ 250 cm^2^ (minimum substrate area required for the smallest crayfish used in this study, ~ 25 mm CL^57^); which offer potential refuge to crayfish. Refuge availability (as cm^2^ m^−2^) was determined by dividing the total area of all cobbles/boulders measured by wetted area of the section. Submerged tree root cavities offer crayfish refuges, but these were infrequent in the current study, so were discounted from refuge availability metrics. Relative crayfish density for each section was determined by dividing the number of crayfish captured through hand-net searching, by the wetted area of the section. Further detail on the hand-searching method and evidence for its suitability is given in Supplementary Information. Although hand-searching incompletely samples the crayfish population, for small streams and when carried out in a standardised way (as here), it provides a sex-unbiased method for density estimation, especially for crayfish > 10 mm CL in size^44^. No significant difference (Mann–Whitney *U* test: *W*= 272, *p* = 0.230) occurred between density estimates obtained from handnet-searching and Surber sampling in the same sections of the study stream. Dewatering for crayfish population surveying^58^, while precise, is inappropriate for in situ ecological studies due to the largescale disturbance that it entails.

### Data analysis

#### Behavioural correlations, consistencies and boldness

Repeatability of behavioural traits over time for recaptured crayfish (activity, distance moved, exploration, climbing, boldness score in newly-established/invasion front) was determined using the package rptR^59^ with 1000 permutations and 1000 bootstraps. To test for evidence of behavioural syndrome between behavioural measures, Spearman’s correlations were performed on the measurements obtained during the first test (i.e. after capture, before release) using Holm–Bonferroni multiple-testing corrections^60^. Differences in boldness, recorded during the threat response test, between the three sites (*n* = 310; fully-established 130, newly-established 90, invasion front 90) were analysed using Fisher’s exact test for a 2 × 3 table.

#### Principal component analysis (PCA)

As the studied behavioural traits were correlated, we performed a PCA with varimax rotation^61^ to define personality trait dimensions^15^ using the R package ‘psych’. Two PCA factors were identified for further analyses based on scree plots and a broken-stick model^62^. As our recaptured sample size was small (*n* = 98), behaviours with a loading of > 0.60 were considered to contribute to the meaning of a component^63^.

#### Factors affecting dispersal in streams

To determine effects of population density on dispersal, we calculated the mean crayfish density in all 5-m long sections traversed (i.e. from the 5-m release section to the 5-m section preceding that in which recapture occurred) by that particular crayfish during dispersal. In streams, crayfish adopt ephemeral home ranges, spending several days at one refuge locality and foraging nearby, before moving to a new refuge locality^42,64^. Refuge habitat for signal crayfish has specific characteristics^42^ and strong competition for refuges can be evident^65^, making daytime refuge use unlikely to be a random process. Dispersal of crayfish in permanent streams is therefore a stepwise process, unlike for example, birds dispersing when they fledge. A similar approach to that described above was used for determination of relationships between dispersal and water depth and also with refuge availability.

We used Generalised Linear Models (GLMs) for combined sites to determine the drivers of dispersal, using type III *F*-tests with the ‘car’ package in R^66^. Global models were generated incorporating site, behavioural trait dimensions (as PCA scores), body mass, population density, habitat characteristics (water depth, refuge availability), missing claw/leg (Yes/No) and sex (M/F) as predictor variables with dispersal rate as the response variable. As we were interested in possible context-dependent variations, interactions of behaviours, refuge and population density with sites were also included in the global model. The global model was subset to select plausible models, based on the ΔAICc values (< 2^67^) using the ‘dredge’ function of the ‘MuMIn’ package in R^68^. We used a model averaging procedure^68^ to generate the final model including all important variables based on model weight of all the models with a ΔAIC < 2. However, we also tested all subset models (ΔAICc values < 2; Table S1) to determine deviation from the ‘final model’ and found similar results (Table S2).

To determine if dispersal is biased towards any specific direction (up/downstream) we used a GLM with dispersal rate as the response variable and dispersal directions as predictors. As data were overdispersed^69^ a negative binomial regression model was employed. There was variation in the number of days crayfish were at liberty, therefore this was added as an ‘offset’ to the GLM, ensuring dispersal was estimated on a standardised scale (m day^–1^). Influence of sex or missing leg/claw on dispersal direction was analysed by using Fisher’s exact test while effect of behaviours (as PCA scores) on dispersal direction was tested by linear regression.

Before analysis, data for body mass, behavioural traits, population density and habitat characteristics were divided by the largest value measured for the sites, which resulted in a proportion for each variable and were normalised to values between zero and one^70^. Variables that contained negative values (e.g. habitat characteristics) were made positive by adding all values with the absolute of the most negative (minimum value) so that the most negative one became zero^71^.

### Ethical statement

Permission for crayfish capture and retention were provided by the Environment Agency (Ref. EP/EW094-U-530/12552/01) and the Fish Health Inspectorate, Cefas (Ref. 020617) respectively. Research approval by Durham University Animal Welfare and Ethical Review Board was made to MCL.

## Results

During recapture surveys in the fully-established site (Westholme) 2659 crayfish were sampled (female: 1225, male: 1175, unsexed small juveniles: 259) of which 41 were marked. In Thorsgill, 1424 crayfish were sampled (female: 628, male: 749, unsexed small juveniles: 47) of which 57 were marked, including 25 individuals from the invasion front and 32 from the newly-established site. There was no detectable difference in sex ratio between the released sample and recaptured samples for fully-established, newly-established and invasion front or combined data (chi-square tests, all *p* > 0.05), although a lower proportion of females were recaptured in fully-established site (Table 2).

### Behavioural consistency and boldness

Among individuals from newly-established and invasion front sites with repeated behavioural tests, all behavioural measures were repeatable over time (*R* = 0.29–0.35; all *p* < 0.004; Table 3). The proportion of crayfish classified as bold or shy based on their responses to the startle test differed between study sites (Fisher exact test, *p* < 0.001). Overall, 53.5% of crayfish were classified as bold (42% at fully-established, 52.2% at newly-established, and 72.2% at invasion front sites).

**Table 3:** Repeatability of behaviours in signal crayfish, measured over time.

Behaviour	Overall			Newly-established			Invasion front		
	*R*	Cl (95%)	*p*	*R*	Cl (95%)	*p*	*R*	Cl (95%)	*p*
Activity	0.35	0–0.68	0.001	0.35	0–0.71	0.001	0.35	0–0.75	0.001
Distance moved	0.34	0–0.69	0.001	0.34	0–0.69	0.001	0.33	0–0.73	0.001
Exploration	0.34	0–0.67	0.001	0.34	0–0.70	0.001	0.34	0–0.75	0.001
Climbing	0.31	0–0.68	0.001	0.31	0–0.71	0.002	0.31	0–0.73	0.002
Boldness	0.29	0–0.64	0.001	0.29	0–0.70	0.004	0.29	0–0.70	0.002

### Correlations between crayfish behaviours—PCA analyses

**Figure 2:**
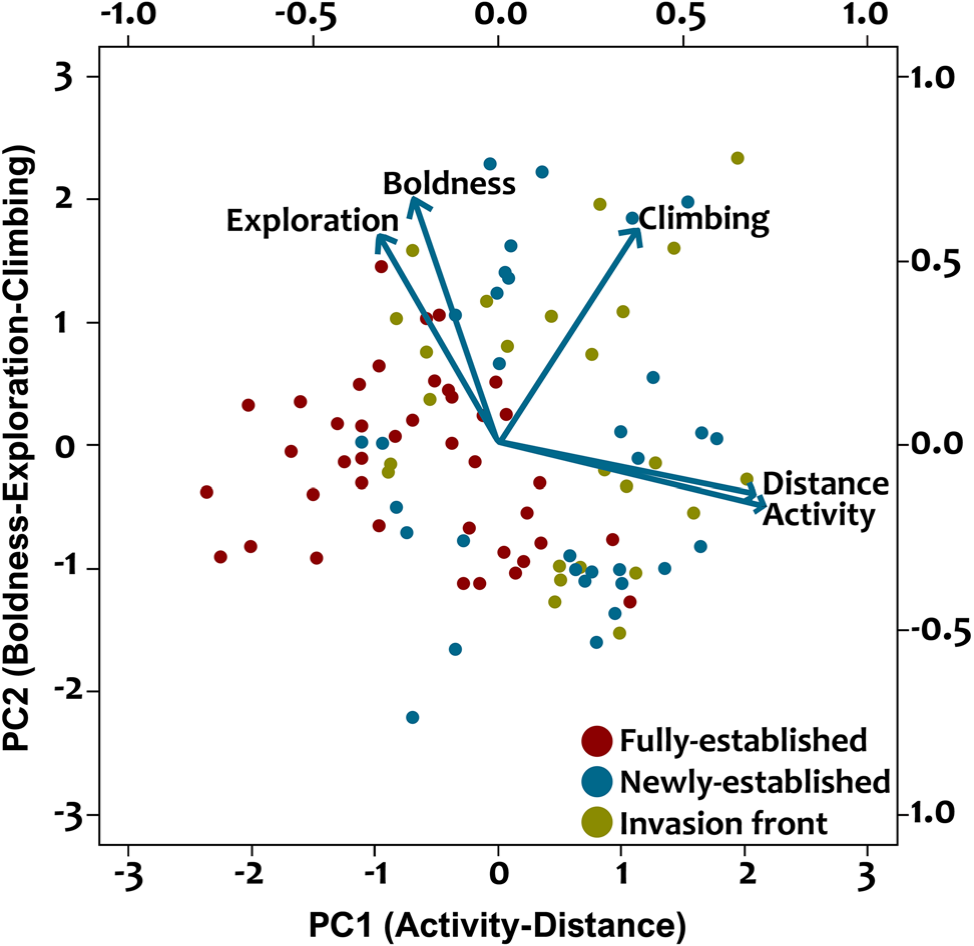
Biplot of principal component analysis with a varimax rotation of the crayfish behaviours. Details of component loadings are given in Table S4. Points represent individual crayfish.

The behaviours measured during the first tests were correlated to each other, except for exploration versus climbing (Table 4, Figure S1). Activity versus distance moved were positively correlated, similar to exploration versus boldness. Notably, activity/distance moved were negatively correlated with exploration/boldness which indicates that more bold and exploratory individuals were less active or moved over a short distance during behaviour assays (Table 4). Climbing duration was positively correlated with activity, distance and boldness. Similar relationships were also observed for measurements taken during the second behavioural test (Table S3). PCA analyses revealed that two axes explained 78% of the variances (Table S4). Activity and distance moved were on PC1 with 0.88–0.90 component loadings, whereas boldness, exploration and climbing were on PC2 with 0.71–0.84 component loadings (Fig. 2, Table S4).

**Table 4:** Spearman’s rank correlations, based on first behavioural test (after capture, before release) of signal crayfish at all sites.

	Activity	Distance	Climbing	Exploration	Boldness
Activity	–	0.76, *p* < **0.001**	0.28, *p* = **0.020**	− 0.47, *p* < **0.001**	− 0.45, *p* < **0.001**
Distance		–	0.24, *p* = **0.040**	− 0.38, *p* < **0.001**	− 0.45, *p* < **0.001**
Climbing			–	0.13, *p* = 0.200	0.25, *p* = **0.040**
Exploration				–	0.71, *p* < **0.001**
Boldness					–

Significant values are in bold.

### Dispersal

Eight recaptured crayfish (one at fully-established site, five at newly-established site, two at invasion front, 8.2% of recaptures) did not move from their original capture site during the study. Dispersal direction was biased upstream slightly (52% of recaptures; site-specific data in Table S5). Mean (± SD and range) dispersal distance for pooled data over the period at liberty was 32.1 ± 27.6 m (0–125 m). Mean upstream dispersal distance in invasion front was 20.6 ± 14.7 m (range: 5–40 m). There was no difference in dispersal rate between upstream (US) and downstream (DS) directions for pooled data (*t*-test, *p* > 0.05; mean ± SD, US 1.1 ± 1.0 m day^−1^; DS 1.3 ± 0.87 m day^−1^). There was no detectable effect of sex or missing claw/leg on dispersal direction (Fisher exact test, all *p* > 0.05).

### Factors affecting dispersal

**Figure 3:**
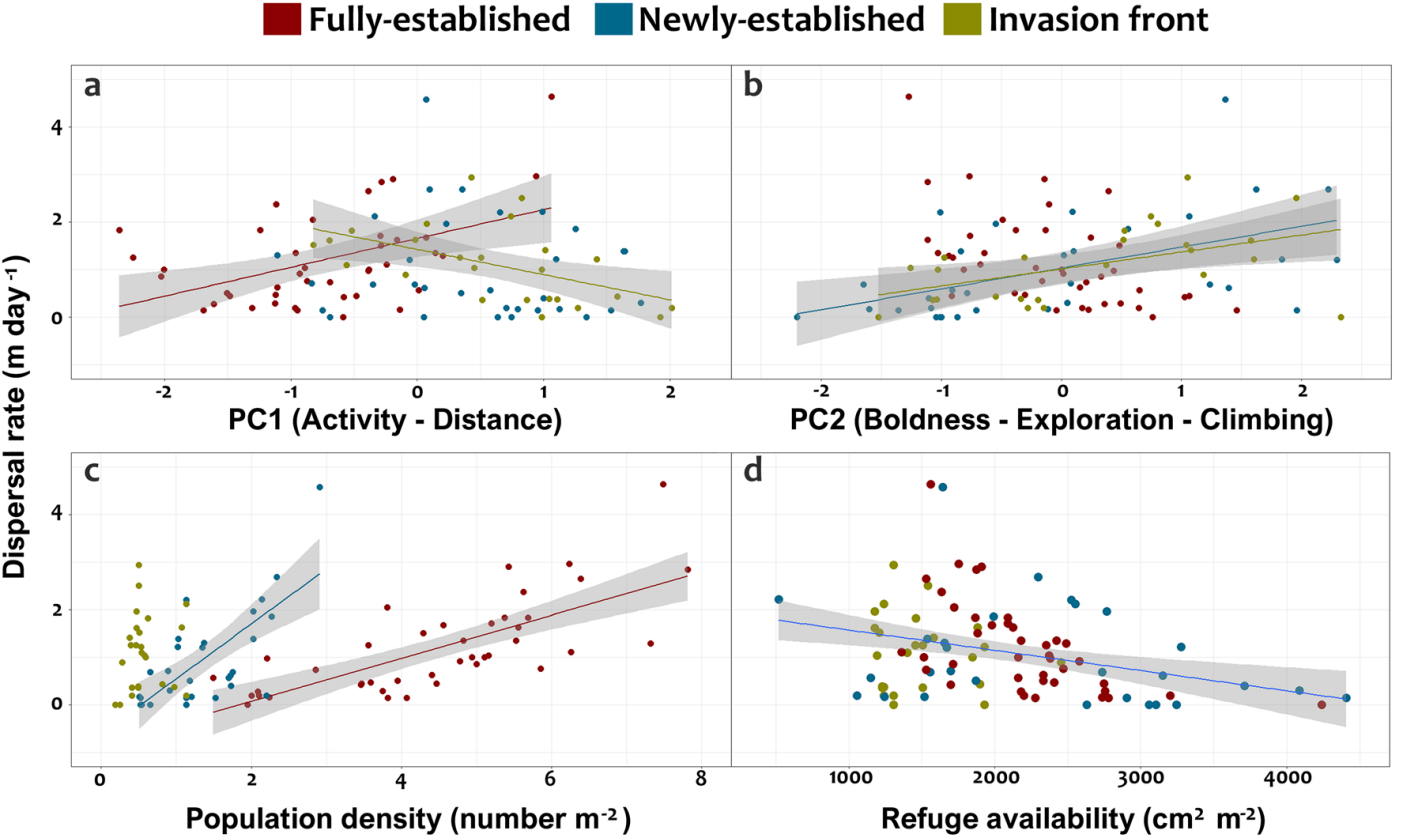
Relationships of behavioural traits, population density and refuge availability to dispersal rate in signal crayfish. Behavioural traits are based on PCA scores whereas mean density and mean refuge represent the average values of crayfish density and refuge of sections traversed during dispersal. Significant relationships (trend lines) and 95% CI are shown.

Dispersal was strongly affected by personality traits, either positively or negatively, at all sites. Dispersal was positively affected by the activity–distance axis at the fully-established site (*p* = 0.021) but negatively at invasion front (*p* < 0.001; Table 5, Fig. 3a). Therefore, individuals behaviourally typed as more active exhibited higher dispersal rates at the fully-established site but lower at the invasion front. On the other hand, there was a positive impact of the boldness–exploration–climbing axis on dispersal rate at newly-established and invasion front sites (Table 5, Fig. 3b). Therefore, bold and exploratory individuals and those that climbed more in behaviour assays, dispersed in the streams at greater rates at these two sites. Faster dispersal was also evident through habitats with high population density (i.e. fully- and newly-established sites; Table 5, Fig. 3c) whereas refuge availability negatively affected the dispersal rate at all sites (Table 5, Fig. 3d). Although dispersal rate was negatively affected by crayfish body mass in two potential models, it was not retained in the final averaged model (Table 5). No effects of other factors (e.g. sex, limb loss and water depth) were found. Dispersal direction was not influenced by behavioural traits (GLMs, *p* > 0.05; Figure S2).

**Table 5:** Final model of factors affecting signal crayfish dispersal rate in relation to personality traits (PC1, Activity-Distance; PC2, Boldness-Exploration-Climbing), population density and refuge availability, obtained by averaging of the top four models shown in Table S3.

Factors	Sum of square	Coefficient estimate	Standard error	*df*	*F*	*p*	95% CI of coefficient
Mass	0.068			1.84	2.72	0.103	− 0.311 to 0.029
Refuge	0.121			1.84	4.83	**0.031**	− 0.485 to − 0.024
**Site**	0.201			2.84	4.01	**0.022**	
FE versus NE		0.024	0.134			0.860	− 0.242 to 0.289
FE versus IF		0.330	0.143			**0.023**	0.047 to 0.614
**Site:PC1**	0.538			3.84	7.15	** < 0.001**	
FE		0.080	0.034			**0.021**	0.012 to 0.147
NE		− 0.022	0.037			0.556	− 0.096 to 0.052
IF		− 0.169	0.043			** < 0.001**	− 0.254 to − 0.083
**Site:PC2**	0.676			3.84	8.99	** < 0.001**	
FE		− 0.035	0.051			0.495	− 0.135 to 0.066
NE		0.089	0.025			** < 0.001**	0.040 to 0.138
IF		0.112	0.030			** < 0.001**	0.051 to 0.172
**Site:Density**	0.577			3.84	7.68	** < 0.001**	
FE		0.548	0.172			**0.002**	0.206 to 0.890
NE		1.347	0.386			** < 0.001**	0.580 to 2.113
IF		1.376	0.984			0.166	− 0.581 to 3.334

Significant values are in bold.

Sites: *FE* fully-established, *NE* newly-established, *IF* invasion front.

## Discussion

**Figure 4:**
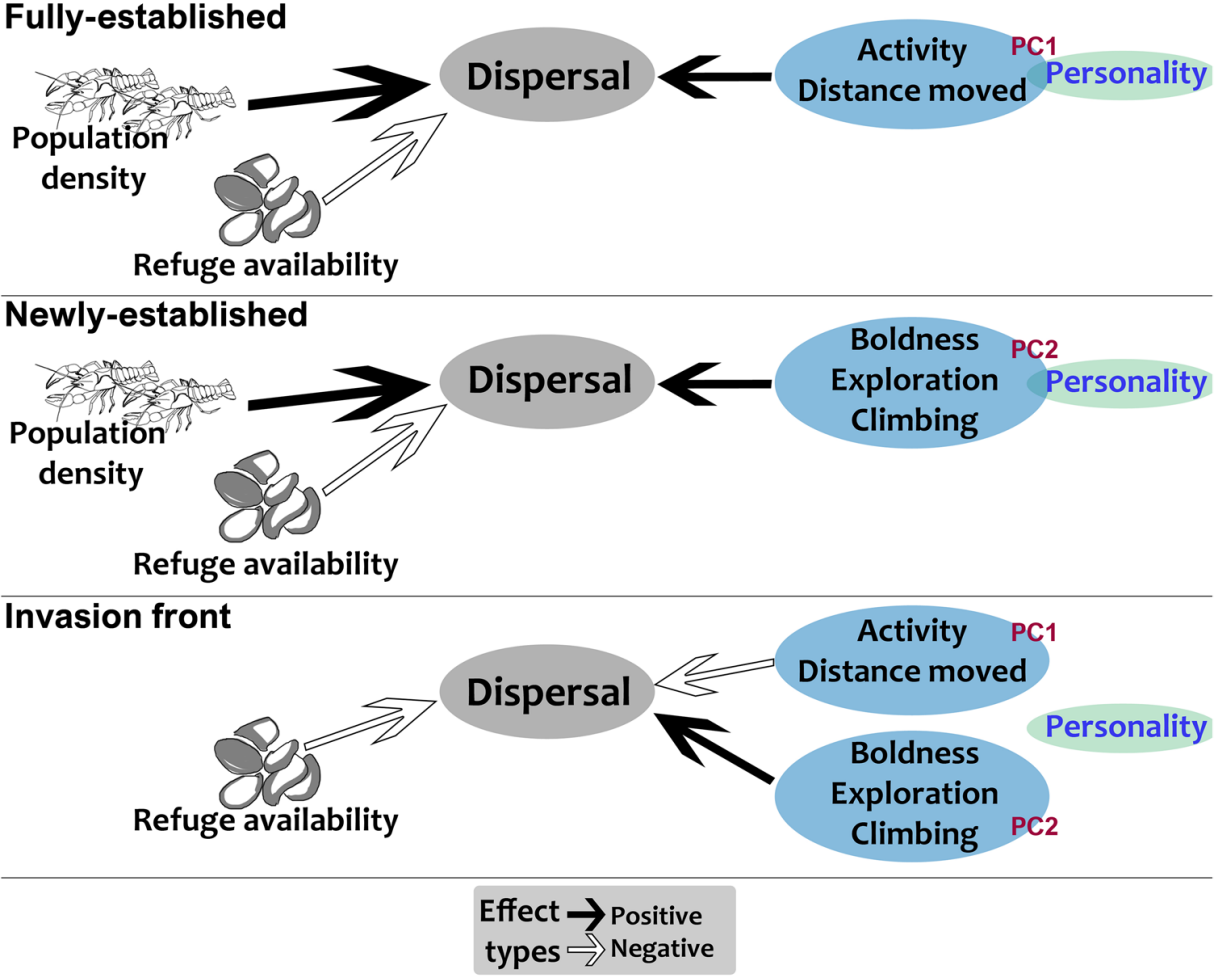
Conceptual diagram showing impacts of personality traits, population density and refuge availability on dispersal of wild signal crayfish at sites differing in terms of invasion stage.

This study provides evidence that dispersal of an invasive species under natural conditions can be driven by individual personality, but that population density and habitat characteristics are important factors also, as hypothesized (Fig. 4). Actively dispersing individuals, including those colonizing streams in an upstream direction, face increased energy expenditure, potentially higher predation risks, and the risk of failing to locate suitable habitat. Understanding the way in which phenotype, including personality, interacts with environment to determine decisions of when to initiate, continue and cease dispersal is necessary to develop a fuller framework of dispersal at the individual scale^19^.

Physico-chemical characteristics of our sites, although uncontrolled, varied little between sites and years and, therefore, are likely to have had negligible influence on the findings. Signal crayfish exhibited individual consistency for focal behaviours over time. We found detectable repeatability in behaviours and combinations of these behavioural traits indicated the existence of behavioural syndromes. However, repeatability values can be low (mean of 0.37 in a meta-analysis^72^) but significant^72^ as recorded in our study (range: 0.29–0.35, all *p* ≤ 0.004). In the field, repeatability values are often lower than laboratory environments^72^, perhaps due to less controlled conditions. A laboratory study with noble crayfish (*Astacus astacus*) showed that boldness can be consistent over time and context^73^. Our study shows that the traits indicative of boldness, along with other behaviours, can be consistent and form a behavioural syndrome and affect dispersal in river networks. Such influences of personality traits on dispersal were predicted for non-native fishes (mosquitofish *Gambusia affinis*^15^, round goby *Neogobius melanostomus*^31^) but not previously demonstrated in the natural environment.

Across study species, personality traits have been shown to have both positive (fish *Rivulus hartii*^74^, bird *Parus major*^75^) and negative (mosquitofish *G. affinis*^15^) impacts on dispersal distance. However, our study provides evidence that the same behavioural traits (i.e. activity-distance moved) can yield different dispersal outcomes for the same species depending upon ecological context, although independent validation of this finding is necessary. In our study, the different contexts comprised sites representing different phases of invasion (Fig. 3). We found a positive relationship between boldness-exploration-climbing and dispersal rate in newly-established and invasion front sites, while activity-distance positively affected dispersal rate at the fully-established site but negatively at the invasion front (Fig. 4). Because of greater crayfish densities at the fully-established site, competition for food and shelter was expected to be higher there compared to newly-established and invasion front sites. At the fully-established site, we hypothesize that shy individuals (exhibiting high activity and distance moved in standardized tests) were likely outcompeted by bolder counterparts and therefore dispersed over relatively longer distances. Conversely, at newly-established and invasion front sites, bolder individuals (low activity and distance moved in tests) dispersed further than shy individuals. Competition for shelter is likely less intense in newly colonised areas and high dispersal rates may not be expected under field conditions for shy crayfish in these areas, as observed in our study, because they spend more time in shelters than bold individuals, even in the absence of predation risk^73^.

In our behavioural assays, without shelters (unlike Vainikka et al.^73^), in which shy crayfish exhibited high activity, especially along tank edges, we interpret this as being due to searching for shelter. This behaviour can be appreciated in the context that crayfish are mostly nocturnal^42,64,65^ and that carrying out behavioural assays by day generates to some degree, behaviours linked to searching for refuges. However, with substantial variation evident between activity and boldness measures (Figure S1) and some studies showing a positive relation between activity and boldness in crayfish^50^ (opposite to our study), hypotheses other than that presented above are tenable. For example, it is possible that more active and shy individuals needed non-refuge resources (more food, for example) for which they dispersed further to a location with more resources but less competition, as observed at the fully-established site, but not at newly-established and invasion front sites where intraspecific competition would be lower. By contrast to the pattern for shy crayfish, bold crayfish are expected to disperse extensively into unoccupied areas, irrespective of shelter^50^.

Although we found an important role for personality traits in determining dispersal rates in signal crayfish, ecological factors were also important determinants. Crayfish dispersal was positively influenced by the local population density in fully- and newly-established sites. Intraspecific competition for food and shelter is expected to be greater in high-density animals populations, generating positive density dependence of dispersal, with dispersers gaining the opportunity to seek better feeding and shelter resources, and increase their fitness prospects^76–78^. In our study, a greater availability of refuges was negatively correlated with higher dispersal rate. Previous studies suggest crayfish distribution is influenced by shelter availability^57,79^ and support the hypothesis of reduced dispersal through habitats with high refuge availability. The retention of mass in two of our subset models (but not the final averaged model), might be attributable to increased tendency to defend local resources against smaller crayfish. Although animal dispersal is often sex-biased (e.g. the invasive bird *Acridotheris tristis*^80^) we did not find any such effect. We found no effect of crayfish limb loss on their dispersal. Although it seems counter-intuitive that missing claws/legs did not affect dispersal^81^, arthropod locomotory ability is not necessarily impeded by loss of one or two walking legs^82^.

Walking animals with greater climbing ability and/or persistence, will be more likely to pass dispersal barriers. Thus, designing effective barriers to prevent non-native species dispersal including invasive crayfish, should consider climbing ability not only from a physiological or kinematic perspective, but also from a behavioural ecology viewpoint. In this study, climbing, a behavioural trait that is not a key focus in most studies, was aligned with the boldness-exploration axis and its effect was substantial in the dispersal of invasive crayfish. A positive relationship between climbing (along with boldness-exploration) and dispersal rate was recorded in newly-established and invasion front sites, which may indicate that climbing behaviour has a significant role in expanding population range through dispersal. A study with juvenile European eel *Anguilla anguilla,* suggests that bold ‘leaders’ which climb test apparatus more readily are more likely to pass real-life obstacles to access upstream nursery habitats^83^. Our results are of particular interest because artificial and natural in-stream obstacles can limit crayfish distribution^65,84^.

Although animal dispersal in many ecosystems, such as land and ocean, may not be directionally constrained, in rivers the channel topography and unidirectional flow have important potential effects on dispersal direction and distance, with resultant implications for demography^85^. We found no influence of behavioural traits on the direction of crayfish dispersal. However, short upstream dispersal at the invasion front indicated a slow upstream range expansion during summer, when signal crayfish are most active^86^. Distribution range expansion rates of 0.96–7.78 m day^−1^ have been recorded for signal crayfish in other studies in streams and rivers^46,87^. In a 19-week long study the majority (82.5%) of signal crayfish moved less than 100 m^88^, similar to the dispersal rate in our study.

Several potential limitations in our experimental approach deserve consideration. First, our study lacks replication of geographically independent invasion fronts. Ecologists agree that it can be difficult to replicate field sites in ecological studies^89^ which was also the case for us. Independent validation of our findings at other invasion fronts is desirable. Second, crayfish are largely nocturnal, so individual responses to the behavioural test may have been different if tested at night instead of during the day. However, previous research suggests that individual boldness is independent of activity chronotype in signal crayfish^48^. Thus it is likely that our boldness indices derived during daylight testing would be similar if conducted at night, although further verification of this would be desirable. A standardised method, used effectively to measure boldness in crayfish^47^ during daylight, was also employed in our study, so any effect of our measurements during daylight would be consistently applicable to all crayfish individuals in our study. Third, this study’s boldness continuum scoring method was based on a standardised threat stimulus that generated behaviours on the defence—retreat behavioural axis^51^, represented by claw raising and tail flipping respectively at opposite ends of this behavioural axis. It could be argued that these two behaviours are distinct and unsuitable for representing as a continuous score. Nevertheless claw raising and tail flipping are established indicators of boldness-shyness^52^ in decapods and a similar scoring pattern is common in crayfish behavioural studies of decapods including crayfish^52,90^. Lastly, we studied the dispersal of signal crayfish for a short time (mean, 34.1 days). However, this was during the season when they are most active^86^ and we speculate that similar phenotypic and environmental effects occur across annual timescales of signal crayfish range expansion (up to 2.4 km of river per year^46^), that reflect the long-term invasion scenario^91^. Based on the comments above, more research is needed to understand the degree to which patterns between dispersal, phenotype and environment in crayfish are repeatable across space and time. We recommend year-long studies at additional invasion field sites, with behavioural trait recording in the dark, and additional boldness measures (e.g. time to emerge after retreat to a standard refuge), to validate the current findings.

To conclude, our study provides evidence that personality and environmental factors influenced dispersal of an invasive animal species. Therefore, understanding the process of biological invasion in animals requires a combined understanding of personality traits, variation within the population and local habitat complexity. Our study highlights the importance of understanding the effects of animal personality as well as environmental factors on the dispersal of animal species; we encourage theoretical and empirical studies that examine and test the interplay between such factors within and across animal taxa, including invasive species.

## Supplementary Information

Supplementary Information.

## Data Availability

The datasets generated during and/or analysed during the current study are available from the corresponding authors on reasonable request.
