# Supplementary material for: Personality, density and habitat drive the dispersal of invasive crayfish
Source: Sci Rep. 2022 Jan 21;12:1114. doi: 10.1038/s41598-021-04228-1 (PMC8782993; doi:10.1038/s41598-021-04228-1)
Supplement: Supplementary file 1 — Supplementary Information. [file 41598_2021_4228_MOESM1_ESM.pdf]

Personality, density and habitat drive the dispersal of invasive crayfish

Shams M. Galib[1,2], Jingrui Sun[1,3,4], Sean D. Twiss[1], Martyn C. Lucas[1]

[1] Department of Biosciences, University of Durham, Durham DH1 3LE, UK

[2] Department of Fisheries, University of Rajshahi, Rajshahi 6205, Bangladesh

[3] Yunnan Key Laboratory of International Rivers and Transboundary Eco-security, Yunnan University, Kunming 650091, China

[4] Institute of International Rivers and Eco-security, Yunnan University, Kunming 650091, China

Supplementary information

Supplementary Methods

Experiments were carried out in summer when dispersal and activity of signal crayfish of both sexes are at their highest[1,2]. In northern England, adult female signal crayfish shed their hatchlings and complete their moult before August. Mating does not commence until early October. The period August-September was chosen for the experiments as a time when most large juvenile and adult crayfish within a population are foraging and dispersing. Dispersal in crayfish does not occur at a discrete time within the lifecycle.

Survey sites

Crayfish are mainly nocturnal and in the shallow streams we sampled, crayfish were very rarely seen in the open, by day (S. Galib, pers. obs.). Therefore, our daytime surveying of crayfish employed sampling of natural shelters for crayfish. The initial density surveys involved effort- and area-standardised random hand-net sampling of potential refuges suitable for juvenile and adult crayfish[3], over 1-km reaches of stream, with 30 minutes of searching at 10 locations (~100 m apart) by two experienced crayfish surveyors. This enabled the upstream invasion front to be located in Thorsgill Beck. Immediately after that, for each of fully-established, newly-established and invasion front sites, six 4–7 m long sections within a ~100 m stretch were searched to estimate the crayfish density. Both streams contained suitable refuges, primarily in the form of unembedded cobbles and boulders, along with infrequent tree roots and burrows for crayfishes (burrows only in Westholme, along a total of ~2% of stream length surveyed). Within study sites there were

no major natural or man-made barriers that could prevent natural dispersal of crayfish upstream or downstream though multiple cascades, riffles, and boulder sills, typical of upland streams, existed. The physico-chemical characteristics of the study sites were similar (Table S1). Assessment of macroinvertebrate populations by standardised kick sampling at four locations ~50 m apart within the fully-established, newly-established and invasion front sites revealed no significant variation in taxonomic richness (Linear Mixed Models, both $p > 0.05$). There were no high-flow events during study periods.

Collection of crayfish for marking

The distance between invasion front and newly-established site centres was 0.5 km and crayfish were collected within the central 45-m and 75-m zones of newly-established and invasion front sites respectively. Signal crayfish with carapace length (CL) <20 mm were not collected as the elastomer tag used in this study (see below) may fragment in smaller individuals[4]. Newly moulted crayfish were also excluded because they tend to remain in their shelters to avoid predation until their exoskeletons harden[5].

Measurement of crayfish abundance and dispersal in natural environment

At the end of the study, all likely, accessible, wetted refuges were hand-net searched thoroughly in contiguous 5-m sections outwards from the midpoints of the zones in which marked crayfish were released. Although we also inspected open streambed for crayfish, all those caught originated from shelters. Although this sampling method contains bias in that, like most crayfish sampling methods [6], the smallest crayfish are undersampled, it provides a standardised, rapid method, effective in shallow upland streams [3]. Unlike trapping, it is relatively non-selective for size and sex and behavioural type [6]. There was a strong positive correlation ($R^2 = 0.76$) in density estimates obtained by handnet-searching and Surber sampling in the same sections of the study stream. No difference (Mann-Whitney $U = 272$, $p = 0.230$) occurred between median density estimates obtained by the two methods. There was no difference in sex ratio either ($U = 338$, $p = 1$), although crayfish < 10 mm CL were consistently under-represented in hand searches. Densities recorded per section are minimum estimates as, inevitably, some crayfish are inaccessible within tree roots or other refuges. On any given day, sampling progressed in an upstream direction to ensure good search visibility and capture efficiency due to disturbed sediment. Surveying occurred outwards from the centre of the reach until no marked crayfish were captured in the outer 300-m zones.

Assessment of physico-chemical parameters and other properties of Westholme and Thorsgill becks

Channel wetted width, calculated as the mean value calculated from three measurements (at 0%, 50% and 100% of length) was taken at each of the 5-m sections, covering a stretch of 1000 m spanning 500 m upstream and downstream from the study sites midpoint. Water depth was recorded at 25%, 50% and 75% of wetted width for each of the 5m sections. This was done for a total of two hundred contiguous 5-m lengths (1 km) of the study reach in each stream w. The flow velocity was measured with an electromagnetic flow meter (Valeport 801, UK) every 50 m (at 50% depth and 25%, 50% and 75% of wetted width) for each 1-km study reach of each stream. Dissolved oxygen (DO), pH, and water temperature were measured bi-weekly (n = 18) from each study site during the study periods. Water level was measured daily at a fixed staff at each study site.

References

1. Bubb, D. H., Lucas, M. C. & Thom, T. J. Winter movements and activity of signal crayfish *Pacifastacus leniusculus* in an upland river, determined by radio telemetry. *Hydrobiologia* **483**, 111–119 (2002).

2. Bubb, D. H., Thom, T. J. & Lucas, M. C. Movement and dispersal of the invasive signal crayfish *Pacifastacus leniusculus* in upland rivers. *Freshw. Biol.* **49**, 357–368 (2004).

3. Bubb, D. H., Thom, T. J. & Lucas, M. C. The within-catchment invasion of the non-indigenous signal crayfish *Pacifastacus leniusculus* (Dana), in upland rivers. *Bull. Français la Pêche la Piscic.* **376–377**, 665–673 (2005).

4. Clark, J. & Kershner, M. Size-dependent effects of visible implant elastomer marking on crayfish (*Orconectes obscurus*) growth, mortality, and tag retention. *Crustaceana* **79**, 275–284 (2006).

5. Helfrich, L. A. & DiStefano, R. J. *Crayfish biodiversity and conservation. Publication No. 420-524*. (2009).

6. Larson, E. R. & Olden, J. D. Field sampling techniques for crayfish. in *Biology and ecology of crayfish* (eds. Longshaw, M. & Stebbing, P.) 287–324 (CRC Press, Taylor and Francis, 2016).

Table S1: Summary of the subset models explaining dispersal rate, yielded from global model. All models with ΔAICc value <2 are included here and examined (Table S3). The summary of the final model is presented in the main text of the paper. PC2, Exploration–Boldness–Climbing, PC1, Activity–Distance moved

**Models**		**df**	**logLik**	**AICc**	**delta**	**weight**
m1	Density+Mass+Refuge+ +PC2+Site+ PC1:Site	11	44.2	–63.4	0	0.341
m2	Density+Mass+Refuge+Site+ PC1:Site+PC2:Site	13	46.7	–63.1	0.32	0.291
m3	Mass+Refuge+Site+ Density:Site+PC1:Site+PC2:Site	15	49.1	–62.3	1.08	0.199
m4	Refuge+Site+ Density:Site+PC1:Site+PC2:Site	14	47.5	–62.0	1.4	0.169

Final model: *Dispersal ~ Mass + Refuge + Site + Site:PC1 + Site:PC2 + Site:Density*

Table S2: Factors affecting dispersal, based on models in Table S1. Model m3 (not shown) is the final model, based on model averaging, and already presented in the main manuscript (Table 5). Sites: FE, fully-established; NE, newly-established; IF, invasion front.

**Model**	**Factor**	**Sum of square**	**Coefficient estimate**	**SE**	***df***	***F***	***P***	**95% CL**
m1	Density	1.16	0.88	0.13	1,88	44.06	**<0.001**	0.61 to 1.14
	Mass	0.14	–0.20	0.09	1,88	5.37	**0.023**	–0.37 to –0.03
	Refuge	0.16	–0.28	0.11	1,88	5.99	**0.016**	–0.51 to 0.05
	PC2	0.67	0.09	0.02	1,88	25.48	**<0.001**	0.06 to 0.13
	Site	0.90			2,88	16.99	**<0.001**	
	FE vs IF		0.57	0.10			**<0.001**	0.37 to 0.76
	FE vs NE		0.30	0.08			**<0.001**	0.15 to 0.45
	PC1:Site	0.56			3,88	7.09	**<0.001**	
	FE		0.07	0.03			**0.048**	0.001 to 0.14
	NE		–0.03	0.04			0.500	–0.10 to 0.05
	IF		–0.17	0.04			**<0.001**	–0.26 to –0.09
m2	Density	0.47	0.69	0.16	1,86	18.38	**<0.001**	0.37 to 1.00
	Mass	0.11	–0.18	0.08	1,86	4.27	**0.042**	-0.34 to -0.01
	Refuge	0.19	–0.31	0.11	1,86	7.37	**0.008**	-0.54 to -0.08
	Site	0.49			2,86	9.51	**<0.001**	
	FE vs IF		0.46	0.11			**<0.001**	0.25 to 0.68
	FE vs NE		0.23	0.08			**0.006**	0.07 to 0.40
	PC1:Site	0.53			3,86	6.81	**<0.001**	
	FE		0.07	0.03			**0.039**	0.004 to 0.14
	NE		–0.03	0.04			**0.489**	-0.10 to 0.05
	IF		–0.17	0.04			**<0.001**	-0.26 to -0.09
	PC2:Site	0.79			3,86	10.21	**<0.001**	
	FE		–0.004	0.05			0.933	-0.10 to 0.09
	NE		0.10	0.02			**<0.001**	0.05 to 0.15
	IF		0.11	0.03			**<0.001**	0.05 to 0.17
m4	Refuge	0.11	–0.24	0.12	1,85	4.35	**0.040**	–0.48 to –0.01
	Site	0.18			2,85	3.58	**0.032**	
	FE vs IF		0.27	0.14			0.056	–0.01 to 0.55
	FE vs NE		–0.04	0.13			0.749	–0.30 to 0.21
	Density:Site	0.57			3,85	7.46	**<0.001**	
	FE		0.50	0.17			**0.005**	0.16 to 0.83

**Model**	**Factor**	**Sum of square**	**Coefficient estimate**	**SE**	***df***	***F***	***P***	**95% CL**
	NE		1.43	0.39			**<0.001**	0.66 to 2.19
	IF		1.42	0.99			0.156	–0.55 to 3.40
	PC1:Site	0.49			3,85	6.44	**0.001**	
	FE		0.08	0.03			**0.019**	0.01 to 0.15
	NE		–0.01	0.04			0.631	–0.09 to 0.06
	IF		–0.16	0.04			**<0.001**	–0.24 to –0.07
	PC2:Site	0.62			3,85	8.04	**<0.001**	
	FE		–0.06	0.05			0.256	–0.15 to 0.04
	NE		0.08	0.02			**0.002**	0.03 to 0.12
	IF		0.11	0.03			**<0.001**	0.05 to 0.17

Table S3: Spearman's rank correlations, based on second behavioural test (after recapture) of signal crayfish at newly-established and invasion front sites.

	**Activity**	**Distance**	**Climbing**	**Exploration**	**Boldness**
Activity	–	0.70; p<**0.001**	0.02; p=0.860	–0.37; p=**0.030**	–0.35; p=**0.040**
Distance		–	–0.14; p=0.570	–0.34; p=**0.010**	–0.38; p<**0.001**
Climbing			–	0.30; p=**0.020**	0.41; p<**0.001**
Exploration				–	0.70; p<**0.001**
Boldness					–

Table S4: Component loadings of crayfish behaviours, obtained through principal component analysis with a varimax rotation. Boldface indicates the highest component loadings for each behaviour.

**Behaviours**	**PC1**	**PC2**
Exploration	–0.40	**0.72**
Activity	**0.90**	–0.18
Boldness	–0.29	**0.84**
Distance	**0.88**	–0.16
Climbing	0.52	**0.71**
Variance explained (%)	42	36
Total variance (%)	78	

Table S5: Summary statistics for dispersal in crayfish sites.

**Dispersal metrics**	**Mean±SD (Range)**		
	**Fully-established (*n*=41)**	**Newly-established (*n*=32)**	**Invasion front (*n*=25)**
Absolute dispersal (m)	34.1±28.1 (0–125)	27.8±28.3 (0–110)	34.2±26.4 (0–100)
Dispersal direction- US (%)	65.9	50	32
Dispersal direction- DS (%)	31.7	34.4	60
Dispersal rate- US (m day^{-1})	1.23±1.01 (0.15–4.63)	1.15±1.16 (0.15–4.58)	0.71±0.49 (0.19–1.25)
Dispersal rate- DS (m day^{-1})	1.22±0.96 (0.14–2.96)	1.26±0.96 (0.17–2.68)	1.45±0.76 (0.36–2.94)

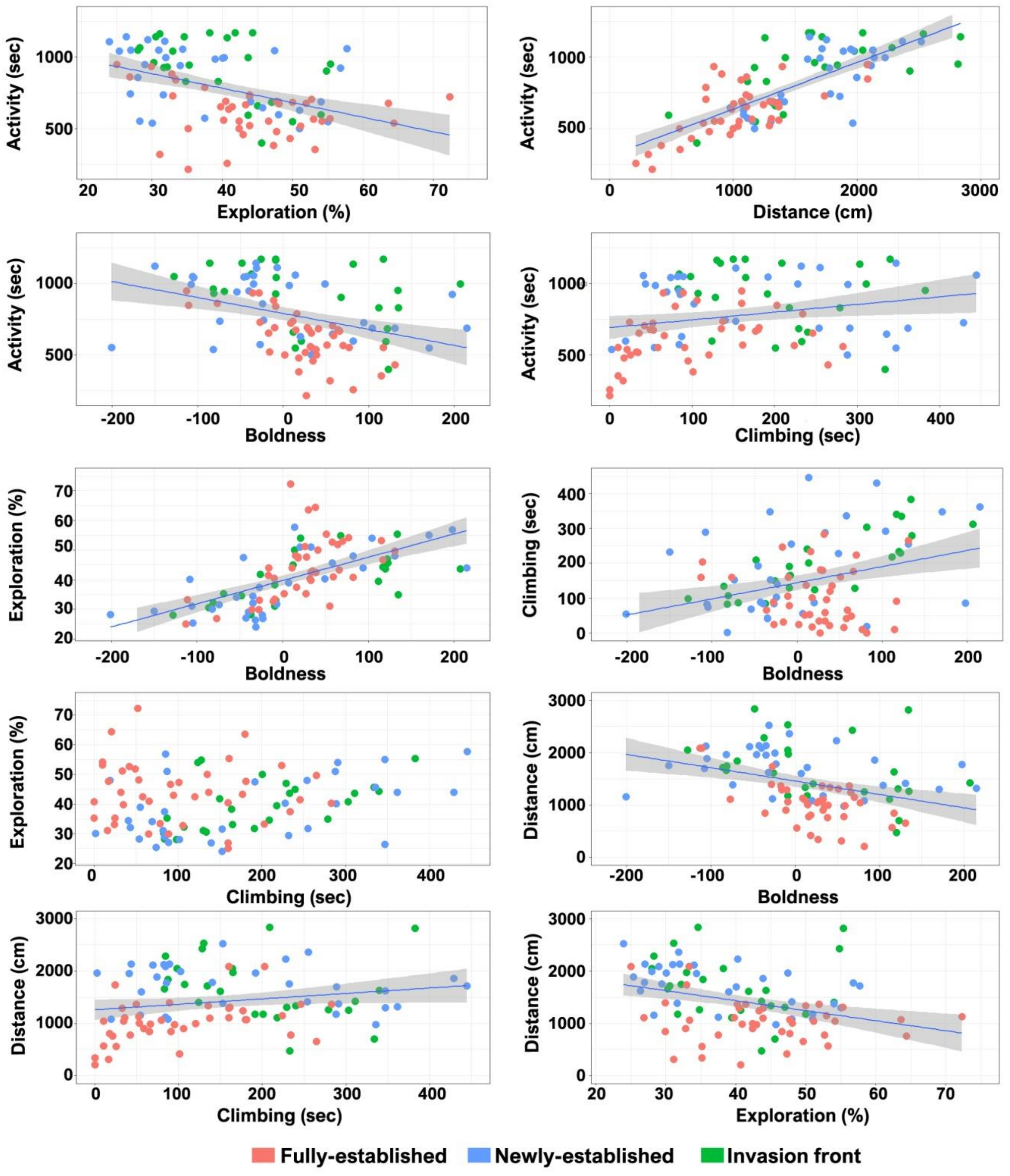

Figure S1: Scatterplots of the correlations between different behavioural traits, in behaviour assays before crayfish release.

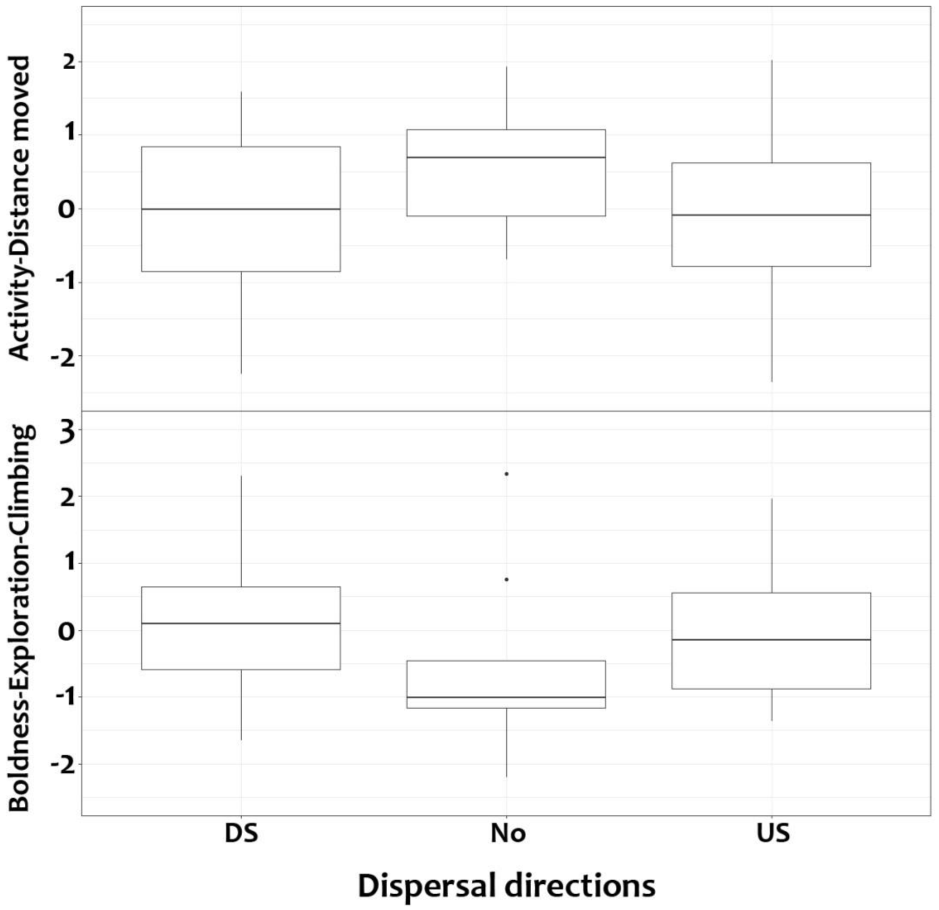

Figure S2: Relationships between behavioural traits (activity-distance moved and boldness-exploration-climbing axes; as PCA scores) and dispersal directions. Midline within the box is the median; upper and lower limits of the box represent the third and first quartile (75th and 25th percentile) respectively.
